# Association between oral findings and laboratory tests in children and adolescents undergoing dialysis: A cross- sectional study

**DOI:** 10.4317/jced.54470

**Published:** 2018-05-01

**Authors:** Aida Esmaeeli, Mohamad Esmaeeli, Masoumeh Ebrahimi, Atefeh Nasehi

**Affiliations:** 1 Dentist,Private Practice, Mashhad, Iran; 2 Associate Professor of Pediatric Nephrology,Mashhad University of Medical Sciences, Mashhad, Iran; 3 Associate Professor of Pediatric Dentistry, Department of Pediatric Dentistry, School of Dentistry, Mashhad University of Medical Sciences, Mashhad, Iran; 4 Dental Research Center, Mashhad University of Medical Sciences, Mashhad, Iran; 5 Assistant Professor of Pedodontics, School of Dentistry, Birjand University of Medical Sciences, Birjand, Iran

## Abstract

**Background:**

Diagnosis of oral complications in the dialysis patients is important to prevent potential infections. The aim of this cross-sectional study was to compare oral findings in dialysis patients with healthy individuals and determination of the correlation of these findings and laboratory tests.

**Material and Methods:**

In this cross-sectional study, DMFT, dmft, DI , CI , OHIS , PI, GI and enamel defects were evaluated in 25 hemodialysis patients, 30 peritoneal dialysis patients and 26 healthy individuals. Then the correlation of laboratory tests (including Hemoglobin, Urea, Creatinine, Ca, Na, Ph, K and ALP) and oral findings was determined in each groups using SPSS (Version 16). Data analyzed with One-way ANOVA test, Chi-Square , Kruskal-Wallis , Tukey’s test and Fisher’s-Exact test.

**Results:**

Findings revealed significant differences in dmft, DI, CI, OHIs, PI and GI between study groups. A positive correlation between Ca and DI was found in hemodialysis group. In peritoneal dialysis group positive significant correlations between DMFT index and Urea, Cr , ALP and K , between OHIs and K , between PI and Cr and negative correlations between Na and CI and OHIs were found.

**Conclusions:**

Presence of oral problems in dialysis patients, especially hemodialysis, indicate the necessity of appropriate therapeutic considerations in these patients. The correlation of blood biochemical compounds and oral status in dialysis patients may warn clinicians to control the level of the biochemical blood compounds for oral health improvement.

** Key words:**Hemodialysis, laboratory tests, oral findings, peritoneal dialysis.

## Introduction

Chronic renal failure (CRF) is caused by chronic progressive destruction of nephrons occurring over several years. While renal failure is usually caused by congenital nephropathy and obstructive uropathy in children under five years, after this age glomerular disease or hereditary disorders are common causes (1). The cause of CRF is unknown in almost 10 percent of patients (2). In patients with CRF several clinical signs and symptoms may be found depending on the degree of the failure of kidney function (1,2). Ninety percent of patients with CRF have oral complications caused by changes in bones and soft tissues of mouth, including metallic taste in the mouth (3), xerostomia (1,4), pale mucous membranes (1,3,4), uremic stomatitis (4), hemorrhage and oral hyperkeratosis, gingival bleeding, petechiae and ecchymosis (2,3), gingivitis, periodontitis, gingival hyperplasia (5), enamel hypoplasia (3), delayed tooth eruption (3,4), malocclusion, calcification of the pulp chamber (4) and temporomandibular disorders (1). Today, treatments of renal failures include drug treatments, specific diet, dialysis (hemodialysis and peritoneal dialysis) and renal transplantation depending on the kidney function and these treatments have satisfactorily reduced oral problems as well as mortality rate of renal diseases (3-5).

The presence of oral infection may act as a focus of infection in other areas of the body in patients undergoing dialysis or transplantation whose immune system has been suppressed (1). Recent studies have shown that oral infections are associated with an increased risk of systemic complications such as atherosclerosis, chronic obstructive pulmonary disease and difficult pregnancy (6). Therefore, the diagnosis of oral complications in the dialysis patients is important to prevent potential infections. On the other hand, as mentioned above, several oral and dental problems can occur due to kidney disease and its medicaments. (7). Overall, according to the available data, the number of patients with CRF in the world is increasing and because of the advancement of pediatric nephrology, the survival rate of these children and therefore referrals to dental clinics have increased (8). Consequently, obtaining the information about oral symptoms and special considerations in these patients is very important to achieve the optimal treatment outcome. Measurement of Urea, Creatinine (Cr), Alkaline phosphatase (ALP), Calcium (Ca), Phosphorus (Ph), Sodium (Na), Potassium (K) and Hemoglobin (Hb) in CBC blood test are some of the characteristic laboratory tests in patients with renal diseases.

Review of available literature indicates that most studies have been done on adult patients with renal disease and there are scare studies on children with CRF. Also, to the best of our knowledge, no study has been done on the relationship between laboratory tests and oral indicators. Therefore, the purpose of this study is to investigate the relationship between oral findings and laboratory tests in children and adolescents undergoing hemodialysis and peritoneal dialysis.

## Material and Methods

In this cross-sectional study, 25 patients undergoing hemodialysis (aged six to 26 years) and 30 peritoneal dialysis patients (aged eight months to 22 years) were selected from a pediatric hospital in Mashhad, Iran, with convenience sampling method. The hemodialysis group (HD) consisted of 13 girls and 12 boys and peritoneal dialysis group (PD) consisted of 13 girls and 17 boys. All patients had end-stage renal failure diagnosed by a nephrologist and were undergoing dialysis in a pediatric hospital in Mashhad, Iran. Twenty-six healthy subjects (without any other systemic disease) with an age range of one to 26 years were recruited from patients referred to Mashhad Dental School. This group, as a control group, consisted of 12 girls and 14 boys and was almost identical to both the study groups in terms of age, gender and social status. The study protocol was approved by the ethics committee of Mashhad University of Medical Sciences, Iran. After a brief explanation of the treatment process an informed consent document was taken from each patient’s parent / legal guardian.

Examination was carried out by an examiner under enough light and with sterile disposable sets including an explorer and an oral mirror as well as WHO probe for periodontal examination. The hemodialysis patients were examined before dialysis and receiving heparin, as receiving heparin may cause prolonged gingival bleeding during BOP evaluation for gingival index.

In each case, first the presence or absence of specific enamel defects was determined. Then caries experience index in permanent (DMFT) and primary teeth (dmft) (9) and oral health assessment indices including Oral Hygiene Index simplified (OHIs) (10), Plaque Index (PI) (11), Debris Index (DI) (10) and GI (12) were studied.

OHI-s comprises Debris Index (DI) and Calculus Index (CI) on six selected tooth surfaces (i.e., buccal surface of the selected upper permanent first molar or second primary molar, lingual surface of the selected lower first permanent molar or second primary molar, labial surface of the upper right and lower left incisors) in six specific teeth. The surface area covered by calculus or debris was determined using an explorer along each tooth surface. The debris scores as well as calculus score were totaled and divided by the number of surfaces scored to measure DI and CI respectively.

Finally, all data related to oral health were recorded in the check list. Then, laboratory tests including Hg, ALP, Ca, Ph, Na, K, Cr and Urea were recorded in all subjects. All blood tests in patients undergoing hemodialysis and peritoneal dialysis were performed in the laboratory of Dr. Sheikh Hospital, Mashhad, Iran. In children aged less than three years, due to lack of cooperation, only dmft, enamel defects and PI (presence or absence of plaque on the buccal surfaces of anterior teeth) were studied. The children were examined only by observation with the naked eye. In the presence or absence of dental plaque score 1 and 2 were given, respectively. The numbers obtained were divided by the number of teeth examined and the result was as PI in each individual.

-Statistical analysis:

For all statistical analysis software SPSS (version 16 ) was used. Kolmogrov-Smirnov was used to verify normality of the data for age. One-way ANOVA was used to compare age in study groups and Chi-Square Test was used to assess distribution of gender in each groups. One-way ANOVA test and Kruskal-Wallis were used to compare the oral finding indices and laboratory tests in the study groups. For the comparison between the two groups, Tukey’s test was used. To assess the frequency of enamel defects in each study groups Fisher’s-Exact test was used. Pearson correlation coefficient was used to determine the relationship between oral findings indices and laboratory tests.

## Results

One-way ANOVA test revealed that there was no significant difference in mean age between study groups (*P*<0.001). Tukey’s test showed that the mean age of patients in the hemodialysis group was significantly higher than peritoneal dialysis (*P*<0.001) and control group (*P* = 0.024), while the mean age in the peritoneal dialysis and control group is not significantly different from each other. Chi-Square Test also showed that the relative frequency distribution of gender among the three groups was not statistically significant.

The One-way ANOVA showed that DMFT among the study groups was not significantly different, but the highest value was observed in the hemodialysis group and the lowest in the control group. Kruskal-Wallis Test showed that dmft was significantly higher in hemodialysis than peritoneal dialysis (*P* = 0.002) and control groups (*P* = 0.016), but showed no significant difference between peritoneal dialysis and control groups.

Comparative evaluation of oral health indices in the study groups showed that DI and GI in the control group were significantly lower than in the hemodialysis group (*P* = 0.032 and *P* = 0. 049 respectively). Both of these variables are also lower in healthy subjects than peritoneal dialysis patients and in the peritoneal dialysis group than in the hemodialysis group, but these differences were not statistically significant. PI was also significantly higher in the hemodialysis group than in peritoneal dialysis and control groups (*P*<0.001). OHI-S in the control group was significantly lower than in peritoneal dialysis (*P* = 0.038) and hemodialysis (*P* = 0.001) groups. However, no significant difference between the hemodialysis and peritoneal dialysis groups was observed. Man-Whitney test revealed that CI in the control group was significantly lower than in peritoneal dialysis (*P* = 0.022) and hemodialysis (*P* = 0.001) groups. However, the difference between hemodialysis and peritoneal dialysis groups was not significant ( Table 1).

**Table 1:**
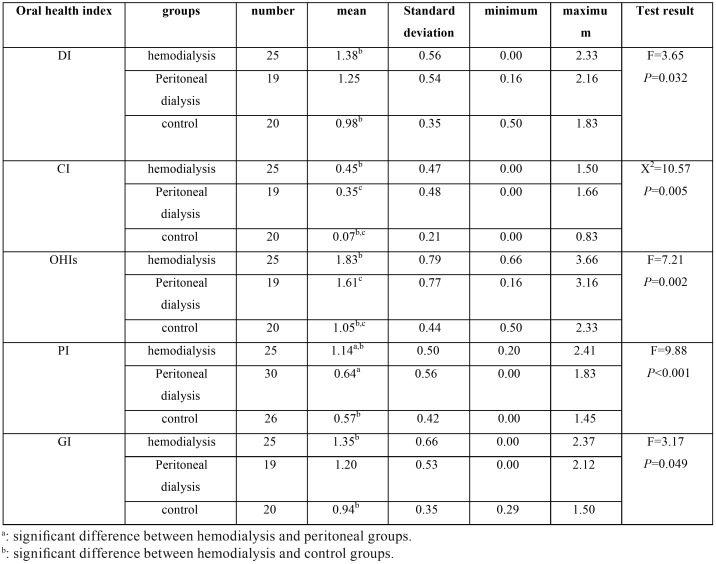
Comparative evaluation of oral health indices in the study groups.

Although 13.3 percent of peritoneal dialysis patients, 8 percent of hemodialysis patients and 3.8 percent of the control group had enamel defects, there was no significant difference between the experimental groups regarding the presence of enamel defects.

A) Association between oral findings and laboratory tests in the hemodialysis group

The results regarding the correlation between caries experience index in primary and permanent dentition and oral health indices with laboratory tests in each of the study groups, after identifying the data for normality and based on the Pearson correlation coefficient (r), are the following:

a. The DMFT and dmft were not correlated significantly with any of the laboratory tests.

b. The oral health indices were poorly correlated with the laboratory tests.

c. Only with increasing the serum Ca, DI was significantly increased (*P* = 0.025) ( Table 2).

**Table 2:**
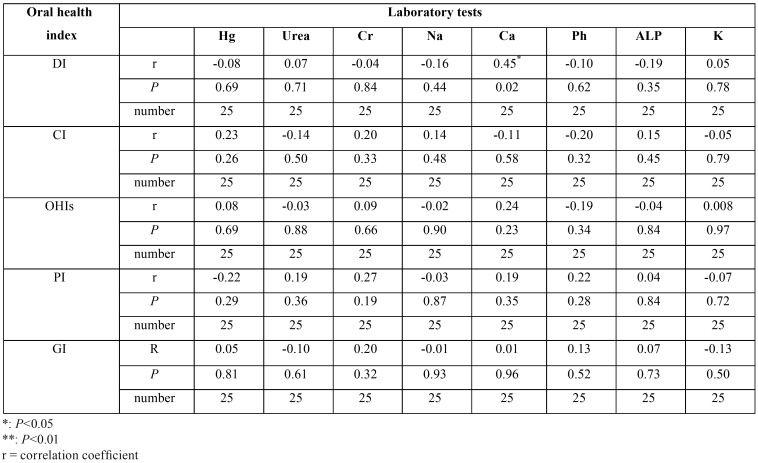
Correlation between oral health indices and laboratory tests in hemodialysis group.

B) Association between oral findings and laboratory tests in the peritoneal dialysis group

a. There is no significant correlation between dmft and none of the laboratory tests.

b. DMFT was significantly increased with increasing in Urea, (*P* = 0.002), Cr, (*P* = 0.024), ALP (*P* = 0.014) and K (*P* = 0.003).

c. CI (P = 0.032) and OHI-S (P = 0.047) significantly decreased with increasing in Na.

d. OHI-S significantly increased with increasing of K(*P* = 0.043).

e. PI also showed a significant increase with increasing in Cr (*P* = 0.004) ( Table 3).

**Table 3:**
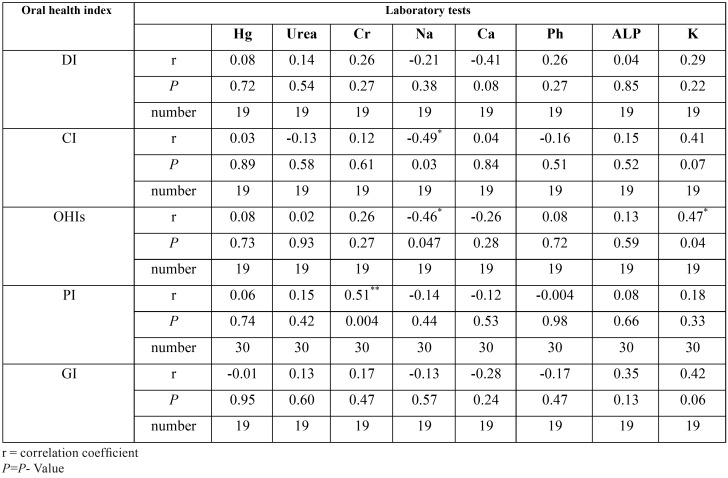
Correlation between oral health indices and laboratory tests in hemodialysis group.

C) Association between oral findings and laboratory tests in the control group

a. Except PI which is significantly increased with increasing of Hb , other oral findings showed no significant correlation with any of the laboratory tests (*P*=0.001) ( Table 4).

**Table 4:**
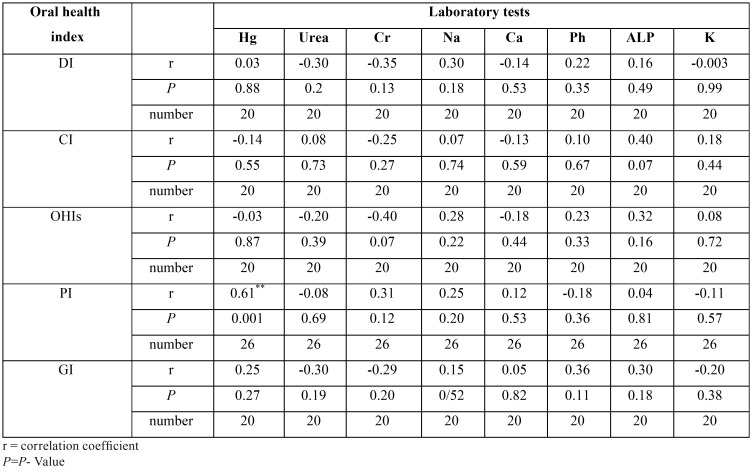
Correlation between oral health indices and laboratory tests in control group.

b. The presence of enamel defects was not significantly associated with any of the laboratory tests.

## Discussion

In the present study, the correlation between oral findings in dialysis patients and blood components were studied. As anemia is one of the signs associated with CRF, hemoglobin was selected and other laboratory tests also revealed the function status of kidney.

Findings revealed that highest value of DMFT and dmft was in the hemodialysis group and the lowest value was in the peritoneal dialysis group. However, these differences were not statistically significant and these findings were in agreement with previous studies (14-16). Low intake of protein is a part of diet of patients undergoing dialysis, and they (especially hemodialysis group), frequently take cariogenic foods, also oral hygiene is poorer than in healthy subjects (17). Consequently, these factors may result in increased DMFT and dmft in dialysis patients. However, no significant difference was found in DMFT index between hemodialysis patients and control group in previous studies and the present study. Considering increase in blood Urea accompanying with increase in salivary Urea, this finding may be the result of inhibitory effect of high salivary Urea in these patients and consequently increasing of the buffering capacity of their saliva (18). According to Jaffe’s study, it was found that increased salivary Phosphate concentration, which is usually seen in dialysis patients, may facilitate the remineralization process of the incipient caries (16). In the current study, we also found a negative correlation between blood Phosphorus and DMFT in the study groups and between blood Phosphorus and dmft in PD patients and control subjects.

In peritoneal dialysis patients, in addition to high Urea and Phosphate in saliva compared to control subjects, quality of life is almost similar to healthy subjects due to less dependence on dialysis centers and it may result in lower value of DMFT and dmft indices in these patients compared with both groups (19).

In the present study, DMFT is decreased by increasing Ca in the hemodialysis group; however, this correlation is not significant. This may be attributed to the role of Ca in enamel maturation and its preservative effect. However, Turtola (20) reported increase in DMFT index associated with increase in salivary Ca and this was in agreement with our findings in peritoneal dialysis and control groups.

We found a decrease in DMFT index with increasing Cr and Ph in all study groups and this correlation was significant in the peritoneal dialysis group (*P* = 0.02, r = 0.53). Increase in Cr was also associated with dmft in hemodialysis and peritoneal dialysis groups in this study. One of the possible causes for a closer and more consonant effect of Cr in oral findings, in comparison to Urea and other blood elements, is the fact that Cr is solely dependent on renal function status. So, in the case of dehydration, due to increased tubular reabsorption, more Urea is reabsorbed in the kidneys, while Cr has no reabsorption in any circumstances. Also, during gastrointestinal bleeding or intake of protein-rich diet, the level of serum Urea increases due to more ammoniac production. Hence, Urea is also influenced by factors other than kidney function (2). To sum up, in all study groups, with the increase in Urea and Cr, dmft is insignificantly increased. This correlation is also found between Cr and DMFT. Therefore, it may be assumed that the serum Cr may have a role in the caries rate.

In present study, findings exhibited that DI was more in HD and PD groups in comparison to control group, and this was in agreement with previous studies (21). Considering the role of saliva in cleansing of debris, the higher value of DI in dialysis groups may be due to dry mouth resulting from CRF.

PI as well as DI showed a significant higher value in hemodialysis patients than in two other groups in the current study. This finding was in agreement with other studies (19,22). The amount of dental plaque is depended on oral hygiene habits and this shows inadequate and low level of individual oral hygiene in end stage renal disease (23). Lower level of PI in peritoneal dialysis patients versus hemodialysis group may imply more attention to oral hygiene in peritoneal dialysis patients. In the current study, PI also increased in all groups with increasing of Cr and ALP. However, this correlation was significant only between PI and Cr. Hence, it appears that the amount of Cr is related to dental plaque; especially in the peritoneal dialysis group. There was also a significant correlation between Cr with DMFT and dmft indices in the study groups.

Similar to previous studies (3,19,21), in the current study, GI was significantly greater in hemodialysis patients than in the control group (*P* = 0.049). However, other studies found different results (16). In these patients, pale tissues resulting from anemia or erythropoietin administration exhibited lower gingival inflammation (24). They also have low oral hygiene and uremic stomatitis and these factors may be the cause of inconsistency in the results of these studies. In the current study, GI and Hb indicated a positive insignificant correlation in hemodialysis with control groups and a negative insignificant correlation in the peritoneal dialysis group. It seems that hemoglobin changes have no effect especially in dialysis patients.

Salivary composition may not completely similar to serum composition. Also, because of probable effect of serum composition on enamel defects, further long-term research with larger sample size is suggested. Besides, evaluation of the correlation of oral findings with salivary biochemical composition in the time of tooth germ formation is recommended.

## Conclusions

As patients undergoing dialysis, especially hemodialysis, have more oral complications than control subjects, the necessity of appropriate treatment considerations and oral hygiene improvement is more predominant in these patients. The correlation of some biochemical blood constituents with oral findings in dialysis patients may help the clinicians in prescribing necessary blood laboratory tests. On the other hand, these correlations warn the physician to balance and control the level of these laboratory tests better than before in order to improve the status of oral health.
